# Cardiovascular magnetic resonance in the German National Cohort and the future of population imaging science

**DOI:** 10.1016/j.jocmr.2025.101963

**Published:** 2025-09-25

**Authors:** Zahra Raisi-Estabragh, Adam J. Lewandowski

**Affiliations:** Centre for Advanced Cardiovascular Imaging, William Harvey Research Institute, Queen Mary University of London, NIHR Barts Biomedical Research Centre, Barts Heart Centre, Barts Health NHS Trust, London, UK; Nuffield Department of Population Health, Big Data Institute, University of Oxford, Old Road Campus, Oxford OX3 7LF, UK; UK Biobank Ltd, Greater Manchester SK3 0SA, UK

Epidemiology is concerned with establishing the distribution and determinants of disease. Reliable causal inference requires large, well-defined cohorts with prospective outcome ascertainment. However, traditional reliance on binary clinical events only captures outcomes once a pre-defined clinical threshold is crossed, ignoring the preceding period where substantial subclinical pathology may be accumulating. This approach reduces sensitivity to early disease states, biases risk estimation, and limits power by dichotomizing complex disease trajectories into late binary outcomes. It also introduces noise from health system factors such as access to care and diagnostic practices, weakening the precision and validity of epidemiological inference.

Cardiovascular imaging provides continuous measures of cardiac anatomy, structure, and function—capturing organ-level alterations before clinically manifest disease. Unlike binary outcomes, imaging biomarkers allow definition of risk across the entire sample and more accurate delineation of disease trajectories, especially in the early and intermediate stages, which are critical windows for disease prevention.

Cardiovascular magnetic resonance (CMR) is the reference standard for assessing cardiac structure and function. It offers unique, non-invasive myocardial characterisation and superior reproducibility compared to other modalities. These strengths make CMR particularly well-suited for large-scale studies, where uniformity in image acquisition is critical for reliable downstream analyses. CMR has been increasingly incorporated into major population-based cohorts such as the Multi-Ethnic Study of Atherosclerosis, the Dallas Heart Study, and the Study of Health in Pomerania. Notably, UK Biobank includes the world’s largest CMR database, having recently completed multi-organ magnetic resonance imaging (MRI) for 100,000 of its participants. Repeat imaging for 60,000 UK Biobank participants is underway, creating an unparalleled resource for longitudinal cardiovascular phenotyping. The emergence of these large-scale cardiovascular imaging datasets has enabled significant advances in biotechnology, image-based epidemiology, and cardiovascular imaging genomics.

The German National Cohort (NAKO) is a landmark epidemiologic study of the German population, integrating large-scale imaging with rich multidimensional data [1]. The study recruited over 205,000 participants aged 19 to 74 years between 2014 and 2019, through a network of 18 examination centers from 16 study regions across Germany. Participants were identified from age and sex stratified samples randomly drawn from compulsory registries of residents within the study regions, with an overall 17% (205,000/1,200,000) response rate. Baseline assessment comprised touch-screen self-report questionnaires, a face-to-face interview, basic physical and medical examinations, and collection of bio-samples, with multiple aliquots stored for future analyses. A random subset of over 56,000 participants completed an enhanced baseline assessment comprising further in-depth physical and medical examinations and self-administered questionnaires. All participants were invited for re-assessment at 5 years from baseline. Whole body MRI was performed in more than 30,000 participants and includes cardiovascular, neurologic, thoracoabdominal, and musculoskeletal imaging [2]. Image acquisition protocols are detailed in dedicated publications [2]. Around 19,000 and 3000 participants have completed a second and third imaging visit, respectively. The collection and processing of data are highly standardized across study centers using pre-defined protocols, equipment, and quality control procedures. Incident health events are ascertained through repeat study assessments, and linkages with registries and insurance databases (some established, others underway). Approved researchers may apply for data access in accordance with the NAKO use and access policy [3].

CMR scans in NAKO are performed in five imaging centers (Augsburg, Berlin, Essen, Mannheim, Neubrandenburg) using 70 cm bore 3.0T MRI scanners (Magnetom Skyra, Siemens Healthineers, Erlangen, Germany) with identical technical features (hardware and software). Scans include standard short- and long-axis cine images acquired using balanced steady-state free precession sequences, parametric T1 mapping, and native angiography covering the pulmonary arterial, venous, and aortic vasculature. Pharmacological stress agents or gadolinium-based contrasts are not administered, given practical and safety considerations in a large research cohort of asymptomatic participants. A dedicated “MRI Core” infrastructure ensures maintenance of high-quality imaging across NAKO imaging centers, long-term storage of image files, integration with non-imaging data, and data distribution to researchers.

Extraction of cardiac phenotypes from large imaging databases requires automated and scalable pipelines for image quality control, anatomic segmentation, and parsing of derived metrics. Full et al. [4] present the first report of the CMR image analysis procedure in NAKO, detailing a meticulous approach to quality control and application of a deep learning segmentation algorithm, previously developed on publicly available datasets [5], to extract left and right ventricular volumetric phenotypes from short-axis images. The authors outline a systematic multi-layered quality control procedure considering selected parameters from morpho-functional, phase differences, and left ventricular time-volume curve analysis to identify likely implausible outlier values using rule-based or statistical thresholds. A procedure is proposed for visual quality control of these potential outliers, and grading of image and segmentation quality in accordance with a standardized scale designed by the authors. The entire workflow is presented, including the number of cases excluded and the reasons for exclusion at each stage. The authors should be commended for the methodological rigor and transparent reporting of their experience of CMR analysis in NAKO, which not only supports researchers who will work with the NAKO resource but also has broad relevance to others who may wish to apply similar standardized image analysis procedures to other imaging datasets.

Thus, NAKO represents an important contribution to epidemiologic and population imaging research, offering high-quality CMR imaging in a large sample presented alongside multimodal baseline phenotyping, repeat assessments, and prospective outcome tracking. The resource presents a platform for testing reproducibility and generalizability of findings across cohorts—relevant to epidemiologic, genomic, and technical studies using CMR. The unique features of NAKO also enable the evaluation of questions not possible using existing resources. The age composition of the sample is an important distinction—with almost 42,000 NAKO participants aged under 40 years old at baseline recruitment, making it one of the largest cohorts of young adults included in a prospective cohort study. Other features, such as differences in exposure profile, disease burden, and factors related to specific local health care systems and policy, enable evaluation of new research questions and cross-comparisons with other large cohorts.

While multi-cohort studies present many opportunities, there are also some challenges. Variation in approach to data collection can lead to systematic biases and limited reliability of cross-cohort comparisons. With regards to CMR, technical variations may be introduced at all stages from image acquisition to post-processing. This may be mitigated at the outset by harmonizing equipment, acquisition parameters, quality control, and image analysis protocols against existing large imaging databanks. In cases where technical variations are unavoidable, transparent reporting of the methods used is essential for understanding the magnitude and direction of differences and may support development of calibration strategies. For instance, if one segmentation algorithm systematically overestimates left ventricular mass, quantifying the degree of deviation allows correction and better interpretability of findings across cohorts. It is also possible to remove post-processing variations in derived phenotypes by retrospective re-analysis of images using uniform post-processing algorithms, facilitating comparison across cohorts and even direct pooling of imaging data. Understanding the impact of technical variations (e.g., segmentation methods, scanner vendor, magnet field strength) can also be helpful for clinical practice, where such variations are common.

Aligning non-imaging variables is often more challenging. Key confounders, such as smoking, alcohol consumption, and physical activity, are frequently collected using different instruments, in ways that are irreconcilable. There may also be important differences in the capture of incident health events, driven by variations in the completeness and quality of outcome documentation, differences in methods of outcome ascertainment, or local variations in health care delivery and diagnostic testing. Transparent reporting of variable definitions and data collection protocols is important to understand these variations and to make informed decisions about their handling and preparation in multi-cohort analyses.

Looking ahead, the future of population imaging science lies in coordinated efforts across large-scale cohorts, enabling discovery through diversity and replication across populations. The next phase of population imaging science will be defined by collaboration, data integration, and methodological innovation. As part of a growing global network of imaging cohorts, NAKO is well-positioned to contribute to harmonized analyses, federated approaches, and the development of robust, generalizable imaging-based biomarkers. Collaboration, standardization, and open access will be central to unlocking the full potential of global imaging resources. NAKO’s scale, standardization, and rich multidimensional data make it an important resource in this evolving landscape.

## Declaration of competing interests

The authors declare that they have no known competing financial interests or personal relationships that could have appeared to influence the work reported in this paper.
